# High-Yield Endoglucanase Production by *Trichoderma harzianum* IOC-3844 Cultivated in Pretreated Sugarcane Mill Byproduct

**DOI:** 10.4061/2010/854526

**Published:** 2010-09-26

**Authors:** Aline Machado de Castro, Marcela Costa Ferreira, Juliana Cunha da Cruz, Kelly Cristina Nascimento Rodrigues Pedro, Daniele Fernandes Carvalho, Selma Gomes Ferreira Leite, Nei Pereira

**Affiliations:** ^1^Renewable Energy Division, Research and Development Center, Petrobras, Avenue Horácio Macedo, 950 Ilha do Fundão, 21941-915 Rio de Janeiro, RJ, Brazil; ^2^School of Chemistry, Federal University of Rio de Janeiro, 21945-970 Rio de Janeiro, RJ, Brazil

## Abstract

The low-cost production of cellulolytic complexes presenting high action at mild conditions and well-balanced cellulase activities is one of the major bottlenecks for the economical viability of the production of cellulosic ethanol. In the present paper, the filamentous fungus *Trichoderma harzianum* IOC-3844 was used for the production of cellulases from a pretreated sugarcane bagasse (namely, cellulignin), by submerged fermentation. This fungal strain produced high contents of endoglucanase activity (6,358 U*·*L^−1^) after 72 hours of process, and further relevant *β*-glucosidase and FPase activities (742 and 445 U*·*L^−1^, resp.). The crude enzyme extract demonstrated appropriate characteristics for its application in cellulose hydrolysis, such as high thermal stability at up to 50°C, accessory xylanase activity, and absence of proteolytic activity towards azocasein. This strain showed, therefore, potential for the production of complete cellulolytic complexes aiming at the saccharification of lignocellulosic materials.

## 1. Introduction

Cellulose is the most abundant polymer on earth, with an estimated amount of 10^12^ metric tons, replenished mainly during plant photosynthesis [1, 2]. In Brazil, which is the main sugarcane producer worldwide [3], one of the most abundant cellulose renewable sources is sugarcane bagasse. In 2008, the Brazilian sugarcane production was 645 million tons [4], generating about 97 million tons of bagasse (dry basis), that is partially used for energy cogeneration at mills [5]. This biomass is composed primarily of cellulose (40%–50%), hemicellulose (25%–35%), and lignin (7%–29%) [6]. 

Cellulases are hydrolases which constitute a complex of several enzymes, which synergistically cleave amorphous and crystalline regions of cellulosic fibers [7]. Such enzymes break down *O*-glycosidic linkages, being classified by the Enzyme Commission with the number 3.2.1.*x*, where *x* varies according to the cellulolytic enzyme. Based on the region of substrate and hydrolysis' products, cellulases can be divided in three main groups [7, 8] the following.

- *Endoglucanases*. This group is represented by the *β*-1,4-endoglucanase (EC 3.2.1.4), which randomly breaks down internal glycosid linkages of the amorphous region of cellulose, releasing polysaccharides with lower degrees of polymerization (DP) than the parent fiber, as well as soluble oligosaccharides (DP < 7);
- *Exoglucanases*. The main enzymes of this group are the cellobiohydrolase (CBH, EC 3.2.1.91) and glucanohydrolase (EC 3.2.1.74). The former, more often reported in literature, catalyzes the split of cellobiose (glucose disaccharide, *β*-1,4-linkage) from either the reducing (CBH type I) or nonreducing (CBH type II) ends of cellulosic fibers, whereas the latter releases directly glucose from the fiber terminals. It is known that both enzymes can be inhibited by their hydrolysis' products.
- *β* *-glucosidases*. The third group of cellulolytic enzymes comprises the *β*-1,4-glucosidase (EC 3.2.1.21), which catalyzes the hydrolysis of cellobiose and soluble oligosaccharides into glucose. This enzyme is also reported regarding its inhibition by glucose.

Cellulases can be used in several industrial applications, such as in textile and laundry, food and feed, pulp and paper, baking, alcohol from biomass, and waste treatment [9–11]. In the textile industry, for example, endoglucanases have been used as an alternative for the abrasive stonewashing process, in eco-friendly technologies, named biopolishing and biostoning, which use, respectively, acid and neutral enzymes [9]. Also, the use of cellulases for ethanol and organic acids synthesis, either by simultaneous saccharification and fermentation (SSF) or by separate hydrolysis and fermentation (SHF) processes, has been extensively reported [12–17]. In these cases, especially in the SSF process, mild temperatures are used, compared to chemical treatments, requiring the use of enzyme complexes that are able to act satisfactorily and retain their activities for several hours at such conditions [18]. 

Therefore, the objective of this work was to evaluate the production of cellulolytic enzymes by the filamentous fungus *Trichoderma harzianum* IOC-3844 from a novel pretreated material derived from an abundant and cheap agroindustrial residue in Brazil, the sugarcane bagasse. In addition, the work aimed at to verify the properties of the enzyme complex produced by this strain in order to prospect its potential applications, with focus on lignocellulose hydrolysis for ethanol production. To our knowledge, this same strain has not been already reported in the open literature for the production of cellulases, thus our findings can contribute to the advance of science in this field.

## 2. Materials and Methods

### 2.1. Sugarcane Bagasse Pretreatments

The pretreated sugarcane bagasse (SCB) evaluated in this work was produced using sequential acid and alkali pretreatments that resulted in the complete removal of hemicellulose and partial removal of lignin fractions [19]. Since the principal remaining components are lignin and cellulose, this material has been named partially—delignified cellulignin, and its cellulose content is 81% higher than that observed in untreated SCB. In a previous work [19], the use of this material as substrate for cellulases production was compared to untreated SCB as well as to SCB submitted only to acid or alkali pretreatments, and up to 9-fold increase in enzyme production was demonstrated when pretreated SCB was used. It represents, therefore, an interesting substrate for cellulases production.

### 2.2. Strain Maintenance

*T. harzianum* IOC-3844 was obtained from the culture collection of Fundação Oswaldo Cruz (Fiocruz), Rio de Janeiro, Brazil. The strain was maintained in PDA plates (DIFCO, New Jersey, USA) at 30°C for 9–10 days before inoculation.

### 2.3. Inoculum and Fermentation Conditions

Resuspended spores of *T. harzianum* (5.33 × 10^7^, total amount) were inoculated in 100 mL of adapted Mandels and Weber medium [20] in 500 mL conical flasks and incubated at 200 rpm and 30°C for 72 hours. Then, 10 mL of the medium containing grown cells (3.35 g · L^−1^) were transferred to 1 L conical flasks containing 200 mL of adapted Mandels and Weber medium two fold concentrated, with glucose substituted by pretreated SCB as carbon source (7.5 g · L^−1^). The systems were incubated at 30°C and 200 rpm. At periodic time intervals, aliquots were withdrawn, sonicated for 1 min for enzymes desorption, and centrifuged at 20,000 × *g* for 5 min., for cells and residual substrate harvesting. Supernatants were stored frozen until analyses.

### 2.4. Assays

FPase, endoglucanase, and *β*-glucosidase activities were determined using Whatman n°1 filter paper, cellobiose, and medium viscosity carboxymethylcellulose (MV CMC, amorphous cellulose, Sigma, St. Louis, USA), as substrates, respectively, according to the standard conditions described by Ghose [21]. These protocols were set as standards for the subsequent analyses. In some experiments, different CMC viscosities were tested to quantify endoglucanase activity, but based on the same methodology cited above. Exoglucanase activity was determined considering the same conditions used for filter paper, just substituting the 1 × 6 cm filter paper strip (equivalent to 50 mg of paper) by 50 mg of avicel CE-15 (microcrystalline cellulose, FMC Biopolymer, Campinas, Brazil). Reducing sugars, expressed as glucose, released during FPase, exoglucanase, and endoglucanase reactions were quantified based on their reducing power towards 3,5-dinitrosalicylic acid (DNS) [22], using glucose as standard for calibration curves. Moreover, glucose released during *β*-glucosidase reaction was quantified using an analysis kit based on the enzymes glucose oxidase and peroxidase (Laborlab, São Paulo, Brazil). *β*-glucosidase activity was also determined incubating 200 *μ*L of crude enzyme extract with 1800 *μ*L of a 3 mM solution (in citrate buffer, pH 4.8) of p-nitrophenyl glucopyranoside (pNPG, Sigma, St. Louis, USA) at 50°C and measuring the initial rate of p-nitrophenol releasing at 405 nm. Endoxylanase and protease activities were determined using Birchwood xylan and azocasein (Sigma, St. Louis, USA) as substrates, according to Bailey et al. [23] and Charney and Tomarelli [24], respectively. All enzyme activities described above, with the exception of protease, were defined as those which release 1 *μ*mol of reducing sugar (FPase, endoglucanase and exoglucanase), glucose (*β*-glucosidase), or p-nitrophenol (*β*-glucosidase) per minute, under the assays conditions. For proteolytic activity, one enzyme unit was defined as the amount of protease that promotes an increase of one absorbance unit (at 345 nm) per minute, under the assay conditions. Total extracellular protein content was measured using the Bio-Rad protein reagent (Bio-Rad Laboratories, Hercules, USA), according to the method described by Bradford [25], and BSA (Sigma, St. Louis, USA) was used as standard. All analyses were done in triplicate in a temperature-controlled incubator (Dubnoff, Nova Técnica, São Paulo, Brazil). For *β*-glucosidase activity measurement using pNPG, a spectrophotometer with a jacketed cuvette chamber (Ultrospec 3100 pro, Amersham Biosciences, Piscataway, USA) coupled with a temperature-controlled bath (ThermoHaake B3, Paramus, USA) was used.

### 2.5. Partial Characterization of Crude Extracts

In order to investigate the dual influence of pH and temperature in the catalytic activity of the cellulolytic enzymes, these factors were manipulated simultaneously. The temperatures evaluated ranged from 30 to 80°C, and the pH values were from 3.0 to 6.0. All analyses were done at standard conditions. Data were fitted to Gaussian and Lorentzian models, and optimal pH and temperature were estimated using the software SigmaPlot version 9.0.

The stability under three temperatures, 37, 50, and 60°C, was determined by incubating the crude extracts in a temperature-controlled incubator during 23 hours. Enzymatic quantifications were done at standard conditions.

For the estimation of *β*-glucosidase parameters, solutions containing 1 to 40 mM of cellobiose were used, whereas for the estimation of endoglucanase parameters, solutions containing from 1 to 20 g · L^−1^ of MV CMC were considered. Reactions were carried out at 50°C, pH 4.8 for 30 min. Parameters were estimated using linear transformations of the Michaelis Menten model, according to Lineweaver-Burk, Eadie-Hofstee, and Hanes plots, as shown in (1)–(3), respectively [26]:

(1) $$\begin{array}{cc} \frac{1}{V} & =\frac{K_{M}}{V_{\max\;}^{\prime }} \end{array}\times \frac{1}{S}+\frac{1}{V_{\max\;}^{\prime }},$$

(2) $$V=V_{\max\;}^{\prime }-K_{M}\times \left(\frac{V}{S}\right),$$

(3) $$\frac{S}{V}=\frac{K_{M}}{V_{\max\;}^{\prime }}+\frac{S}{V_{\max\;}^{\prime }}.$$

Finally, the catalytic spectrum in several substrates was examined for the cellulolytic extract from *T. harzianum* and compared to those of two commercial preparations, which were kindly provided by their suppliers: Celluclast^®^ (Novozymes, Araucaria, Brazil) and Spezyme^®^ (Genencor International Inc., Rochester, USA).

## 3. Results and Discussion

### 3.1. Cellulases Production Using Pretreated SCB

**Figure 1:**
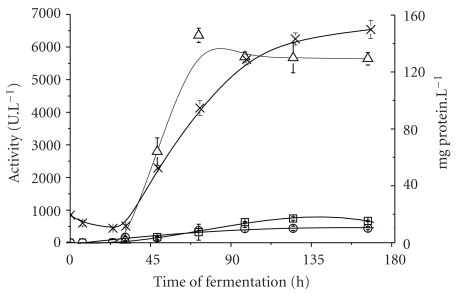
Cellulases production by *T. harzianum* IOC-3844. (-◯-) FPase activity; (-□-)  *β*-glucosidase activity; (-△-) Endoglucanase activity; (- × -) Protein content.

*T. harzianum* was cultivated in conical flasks by SmF in the presence of pretreated SCB and nutrients in order to induce high-level cellulases production. SCB was tailoredly pretreated in order to generate a high-cellulose-content material (69% glucan) with improved fiber exposition to microbial attack. Recently, when compared to other sugarcane bagasse-derived materials, pretreated SCB induced the best the production of cellulases by *Penicillium funiculosum* [19].  Figure 1 shows kinetic profiles for the production of three groups of cellulolytic activities, as well as the total extracellular protein content of the extracts, by *T. harzianum*. 

*T. harzianum* showed the ability for an outstanding production of endoglucanase activity, represented by fast kinetics when compared to FPase and *β*-glucosidase, reaching a maximum activity of 6,358 U · L^−1^ for the former. The exponential production phase for endoglucanase was detected between 31 and 72 h of fermentation, after a short lag phase. Although it was not the most marked activity, *β*-glucosidase production by *T. harzianum* IOC-3844 (742 U · L^−1^ after 125 h of fermentation) can be also considered satisfactory, being a little higher than that reported by Ahmed et al. [27], after their study of the cultivation of *T. harzianum* E-58 (629 U · L^−1^). 

In terms of volumetric productivity and specific activity, *T. harzianum* proved to produce high titers of cellulases, even when compared to the model and widespread fungus *Trichoderma reesei* Rut C30. In addition, the potential of the fungi *Penicillium funiculosum *ATCC11797 and *T. harzianum* IOC-4038, all of them cultivated in the same material, the pretreated SCB, was also compared (Table 1). The comparison indicated that the strain *T. harzianum* IOC-3844 is a promising cellulases producer when a pretreated material from a cheap and highly available biomass in Brazil is used. Although *T. reesei* Rut C30 has been recognized for decades as a strain which produces hypercellulolytic complexes [29], its performance onto lignocellulosic carbon sources is not outstanding as it is under pure cellulosic substrates [30, 31]. Longer acclimation periods observed when this strain is cultivated in lignocellulosic feedstocks, when compared to pure carbon sources [32], can be due to the several genetic mutations that it was submitted [33, 34] as well as deficiency in its enzymatic machinery capable to degrade lignin [35], which is more characteristic of soft rot fungi [36]. Moreover, the use of partially delignified biomasses has been reported to either positively or negatively influence cellulases production by *T. reesei* strains, depending on the alkali pretreatment used [37, 38]. Further studies made in our laboratories proved that when cultivated in the presence of pure cellulosic sources, such as CMC, avicel, and cellobiose, *T. reesei* Rut C30 produced cellulolytic extracts four- to fivefold concentrated when compared to those obtained in pretreated SCB [32].

### 3.2. Dual Investigation of Temperature and pH Influence on Cellulolytic Activities

**Figure 2:**
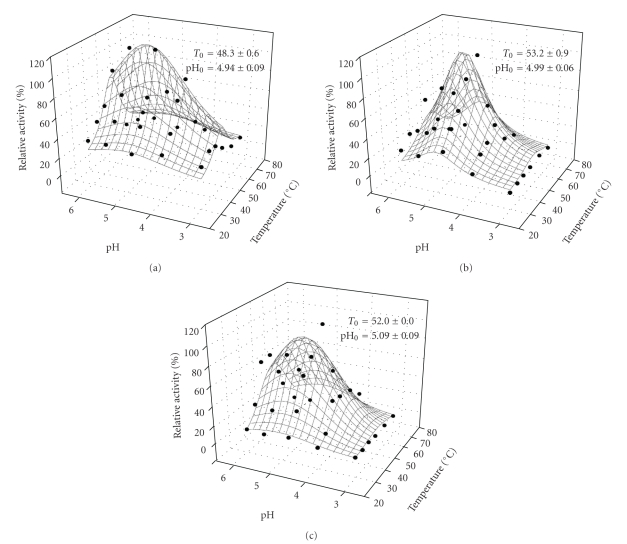
The effect of pH and temperature on enzymatic activity of (a) FPase, (b) *β*-glucosidase, and (c) endoglucanase activities from *T. harzianum*. Maximum activities, observed at the optimal temperature (*T*_o_), and pH (pH_o_) were 1,143; 885 and 21,079 U · L^−1^, respectively.

The effects of temperature in the range from 30 to 80°C and pH in the range from 3.0 to 6.0 were investigated in the three standard enzymatic quantifications: FPase, endoglucanase, and *β*-glucosidase. 3D graphs showing the dual effect of these parameters in the activities of cellulases from *T. harzianum* are presented in Figure 2. *β*-glucosidase and FPase plots were fitted to Lorentzian model, whereas endoglucanase data were adjusted according to Gaussian model. All of the groups of enzymes evaluated presented their highest catalytic power at similar conditions (around 50°C and pH 5.0), which is desired, for example, when a complete cellulose hydrolysis is aimed, since in this condition their synergistic action towards the substrate is maximized. The optimal conditions observed for the cellulases produced by *T. harzianum* IOC-3844 are in accordance with the usually observed for fungal cellulases from mesophilic strains and fit very well to SHF processes [39].

Besides, at suitable conditions for SSF processes (commonly 37°C and pH 5.5) [17], the experimental relative activities of FPase, endoglucanase, and *β*-glucosidase produced by *T. harzianum* were, respectively, 35.9 ± 4.2, 23.6 ± 2.4, and 34.9 ± 0.3, indicating that the crude cellulolytic extract produced by this fungus presents a potential for ethanol and other chemicals production using processes that require hydrolysis at mild conditions.

### 3.3. Thermal Stability of Crude Cellulolytic Complex at Various Temperatures

**Figure 3:**
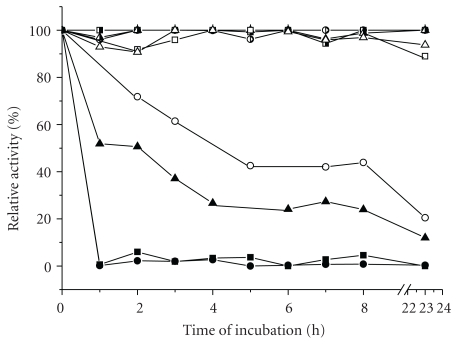
Thermal stability of FPase (triangles), endoglucanase (squares), and *β*-glucosidase (circles) produced by *T. harzianum* at 37°C (black and white symbols), 50°C (white symbols), and 60°C (black symbols).

The investigation of stability of the enzymes produced by SmF of *T. harzianum* at three temperatures was carried out during 23 h, which is a period enough or even longer to reach a stationary phase for ethanol production in SSF processes [16, 17]. Profiles of relative enzymatic activity are presented in Figure 3. As expected, the higher the temperature, the higher the loss in activity. *β*-glucosidase activity was the most sensitive to the two highest incubation temperatures, 50 and 60°C, presenting half life times of about 4 h and less than 1 h, respectively. At 37°C all enzymes produced by *T. harzianum* maintained at least 90% of their initial activities after 23 h of incubation.

### 3.4. Estimation of Kinetic Parameters of *T. harzianum* Cellulases

Estimation of the apparent kinetic parameters, *K_M_* and *V*′_max _, was done for endoglucanase and *β*-glucosidase activities in the crude extract produced by *T. harzianum*. FPase activity was not considered in this case, since the substrate is insoluble and several further effects would influence the catalysis in heterogeneous conditions, thus the behavior tends to diverge from the Michaelis Menten model. Results obtained by fitting the experimental observations during the initial rate (first-order region) are shown in Table 2. Although Lineweaver-Burk (also known as the double reciprocal plot) is possibly the most reported one, other two linear models were evaluated, in order to compare their results. 

It can be observed that Lineweaver-Burk and Eadie-Hofstee models returned similar values for *K_M_* of both endoglucanase and *β*-glucosidase activities, while Hanes model indicated lower affinity of the former group of enzymes towards CMC and higher affinity of the latter group for cellobiose. Regarding the maximum apparent rate of hydrolysis, the fittings were quite different between the two groups of enzymes, which can be explained by the distinct accuracy of these models. While Lineweaver-Burk model usually overemphasizes data obtained at low substrate concentrations, in Eadie-Hofstee model the dependent variable* V *(initial rate of hydrolysis observed for each substrate concentration) appears in both coordinates of its plot, leading to error propagation. According to Leskovac [26], the Hanes model, thus, seems to be the most accurate for estimation of kinetic parameters.

### 3.5. Substrate Specificity of the Enzymes from *T. harzianum*

The crude final extract obtained by culturing *T. harzianum* in pretreated SCB was incubated with several substrates, in order to determine the specificity of its enzymes over different structures. The detected activities are presented in Table 3, besides the correspondent activities of two commercial preparations. It can be observed that the commercial products presented activities from 100- to 500-fold higher than the crude extract produced by *T. harzianum*, which is expected due to purification and concentration steps subjected to large-scale process streams, such as membrane ultrafiltration [40]. Undesired proteolytic activity was not detected in *T. harzianum* extract, as well as in commercial preparations. It is known that proteases can affect enzyme stability [41] and that the glycosylation extent, that can correspond up to 39% of total cellulase mass [42], may contribute to protease resistance [43]. Xylanase activity was detected in both *T. harzianum* extract and commercial products, despite the absence of xylan in the raw material used in the fermentations. This activity, on the other hand, is desired in the extract, since such accessory enzyme can contribute to enhanced cellulose hydrolysis, acting synergistically with cellulases [44].

Concerning the three distinct CMC sources used in this evaluations, it can be observed that the higher the viscosity of the solutions, the lower the activity, which is probably due to diffusivity limitations. Similar behavior was detected by Castro et al. [19], regarding the cellulolytic extract produced by *P. funiculosum*. The performance observed for endoglucanase activity from* T. harzianum* suggests that throughout the hydrolysis of cellulosic sources, as the DP is decreased, the viscosity of solutions is decreased and endoglucanases may have their activities improved, when compared to the beginning of hydrolysis.

Considering the three major groups of cellulases, that are *β*-glucosidase, endoglucanase, and exoglucanase, represented in this work by their action in cellobiose, MV CMC, and avicel, respectively, and their proportionality to the activity towards filter paper (FPase), some observations can be done. Inferring a unitary normalized activity for FPase and associating it to the activities of *β*-glucosidase, endoglucanase, and exoglucanase in the extract produced by *T. harzianum*, it can be found a proportion of 1 : 0.41 : 8.5 : 0.59. The product Celluclast^®^ (proportion 1 : 0.72 : 20.9 : 0.60) presented similar balance between FPase and exoglucanase, but higher contribution of *β*-glucosidase activity in the overall complex, which is desired for the complete cellulose hydrolysis. However, the very high proportion of endoglucanase activity in this preparation, when compared to FPase, indicates that the action of its enzymes is quite better in soluble than in insoluble cellulosic substrates. The preparation Spezyme, on the other hand, showed more ability for the hydrolysis of insoluble cellulosic source (filter paper) than the soluble one (MV CMC), but deficient activity of end-acting enzymes (exoglucanase and *β*-glucosidase), representing a great disadvantage for SSF processes. 

Therefore, the enzyme complex produced by *T. harzianum* from a sugarcane bagasse cellulignin demonstrated to have well-balanced activity between soluble and insoluble cellulosic sources, which is desired because of their whole action during saccharification processes (SSF or SHF) of agroindustrial materials. At the beginning of such processes, the cellulosic source is commonly insoluble (crystalline and amorphous fibers with high DP). As the hydrolysis occurs, the material is liquefied into oligosaccharides, so soluble molecules become preponderant in the medium [45], requiring enzymes (still endoenzymes) that can release smaller oligosaccharides and cellobiose for the synergistic action of *β*-glucosidase, and the final release of glucose.

The cellulolytic complex produced by *T. harzianum*, in addition to its high endoglucanase content, presented adequate characteristics for its application in cellulose hydrolysis. It can be used, therefore, in saccharification processes aiming at ethanol and other chemicals production.

## 4. Conclusions

The filamentous fungus *Trichoderma harzianum* IOC-3844 was studied regarding its potential for the production of cellulolytic enzymes. When cultivated in a sugarcane bagasse-derived material containing high cellulose content (69% glucan), *T. harzianum* produced 6,358 U · L^−1^ of endoglucanase activity and also significant levels of *β*-glucosidase and FPase. The enzymes showed to be best active at temperatures around 50°C and acid pH values (5.0) and proved their thermal stability at conditions suitable for an SSF process. Based on comparison with commercial preparations, the extract from *T. harzianum* presented well-balanced cellulolytic activities towards insoluble and soluble substrates, indicating that this action in an SSF process is able to occur during the whole process. *T. harzianum* IOC-3844 is, therefore, a promising strain for cellulases production by SmF using a pretreated raw material derived from a low-cost agroindustrial byproduct.

## Figures and Tables

**Table 1:** Maximum volumetric productivity and specific activity values for cellulases production by SmF of *T. harzianum* and related literature using pretreated SCB as substrate. Values in parentheses correspond to times of fermentation (h) when the values were observed.

Microorganism	FPase	Endoglucanase	*β*-glucosidase	Reference
Volumetric productivity (U · L^−1^ · h^−1^)				

*T. reesei* RutC30	0.05 ± 0.02 (333)	0.31 ± 0.03 (333)	0.25 ± 0.01 (333)	[28]
*P. funiculosum*	2.83 ± 0.46 (72)	16.76 ± 0.91 (100)	8.54 ± 0.35 (100)	[19]
*T. harzianum* IOC-4038	1.10 ± 0.10 (71)	7.60 ± 0.36 (32)	9.57 ± 0.41 (71)	[28]
*T. harzianum* IOC-3844	5.33 ± 0.82 (31)	88.41 ± 3.05 (72)	6.46 ± 0.17 (98)	This paper

Specific activity (U·(mg·protein)^−1^)				

*T. reesei* RutC30	0.28 ± 0.10 (333)	1.64 ± 0.15 (333)	1.34 ± 0.07 (333)	[28]
*P. funiculosum*	7.98 ± 0.07 (49)	43.14 ± 4.22 (49)	19.06 ± 0.47 (49)	[19]
*T. harzianum* IOC-4038	0.92 ± 0.11 (120)	6.42 ± 0.31 (32)	7.09 ± 0.13 (120)	[28]
*T. harzianum* IOC-3844	14.00 ± 2.16 (31)	67.14 ± 2.32 (72)	5.30 ± 0.35 (125)	This paper

**Table 2:** Kinetic parameters of endoglucanase and *β*-glucosidase from *T. harzianum* determined by different linear models.

Kinetic parameters	Endoglucanase	*β*-glucosidase
*K*_*M*_^a^		

Lineweaver-Burk	19.39	0.5829
Eadie-Hofstee	19.51	0.6298
Hanes	23.95	0.3639

*V*′ _max _ (mmo$\text{l}\cdot {\text{L}}^{-1}\cdot \overset{-1}{\min\;}\;$)		

Lineweaver-Burk	0.0948	0.0292
Eadie-Hofstee	0.0219	0.0305
Hanes	0.1470	0.0275

^a^For endoglucanase, expressed as g · L^−1^ and for *β*-glucosidase, expressed as mM.

**Table 3:** Substrate specificity of the crude cellulolytic extract from *T. harzianum* and its comparison with commercial preparations.

Substrate	Activity (U · L^−1^)		
	*T. harzianum*	Spezyme^®^	Celluclast^®^
Avicel	(5.12 ± 0.28) × 10^2^	(1.14 ± 0.14) × 10^5^	(3.10 ± 0.20) × 10^4^
Azocasein	ND	ND	ND
Cellobiose	(3.60 ± 0.36) × 10^2^	(2.90 ± 0.20) × 10^4^	(3.70 ± 0.40) × 10^4^
HV CMC	(3.98 ± 0.09) × 10^3^	(1.26 ± 0.06) × 10^6^	(8.67 ± 0.25) × 10^5^
MV CMC	(7.37 ± 0.94) × 10^3^	(1.83 ± 0.16) × 10^6^	(1.07 ± 0.01) × 10^6^
ULV CMC	(1.20 ± 0.08) × 10^4^	(2.42 ± 0.01) × 10^6^	(1.32 ± 0.09) × 10^6^
Filter paper	(8.71 ± 0.32) × 10^2^	(3.83 ± 0.67) × 10^5^	(5.10 ± 0.80) × 10^4^
pNPG	(1.13 ± 0.02) × 10^3^	(1.10 ± 0.02) × 10^5^	(5.40 ± 0.10) × 10^4^
Birchwood xylan	(3.93 ± 0.03) × 10^2^	(8.15 ± 0.30) × 10^5^	(4.40 ± 0.14) × 10^5^

For abbreviations, see List of Abbreviations.

## Abbreviations

CMC: Carboxymethylcellulose
DP: Degree of polymerization
FPase: Filter paper hydrolase
HV: High viscosity
*K_M_*: Apparent Michaelis constant
MV: Medium viscosity
ND: Activity not detected
PDA: Potato-dextrose-agar
pNPG: p-nitrophenyl glucopyranoside
*S*: Substrate concentration
SCB: Sugarcane bagasse
SHF: Separate hydrolysis and fermentation
SmF: Submerged fermentation
SSF: Simultaneous saccharification and fermentation
ULV: Ultra low viscosity
*V*: Apparent reaction rate at certain substrate concentration
*V* ′_max _: Apparent maximum reaction rate.
